# A Shiga Toxin-Encoding Prophage Recombination Event Confounds the Phylogenetic Relationship Between Two Isolates of *Escherichia coli* O157:H7 From the Same Patient

**DOI:** 10.3389/fmicb.2020.588769

**Published:** 2020-10-23

**Authors:** David R. Greig, Claire Jenkins, Timothy J. Dallman

**Affiliations:** ^1^National Infection Service, Public Health England, London, United Kingdom; ^2^Division of Infection and Immunity, The Roslin Institute and Royal (Dick) School of Veterinary Studies, University of Edinburgh, Easter Bush, United Kingdom

**Keywords:** Shiga toxin-producing *E. coli*, genomics, Nanopore, Prophage, recombination, relatedness

## Abstract

We compared genomes from multiple isolations of Shiga toxin-producing *Escherichia coli* (STEC) O157:H7 from the same patient, in cases notified to Public Health England (PHE) between 2015 and 2019. There were 261 cases where multiple isolates were sequenced from the same patient comprising 589 isolates. Serial isolates from the same patient fell within five single nucleotide polymorphisms (SNPs) of each other for 260/261 (99.6%) of the cases, indicating that there was little evidence of within host variation. The investigation into the 13 SNP discrepancy between one isolate pair revealed the cause to be a recombination event within a *stx2a*-encoding prophage resulting in the insertion/deletion of a fragment of the genome. This 50 kbp prophage fragment was homologous to a prophage in the reference genome, and the short reads from the isolate that had the 50 kbp fragment, mapped unambiguously to this region. The discrepant variants in the isolate without the 50 kbp fragment were attributed to ambiguous mapping of the short reads from other prophage regions to the 50 kbp fragment in the reference genome. Identification of such recombination events in this dataset appeared to be rare, most likely because the majority of prophage regions in the Sakai reference genome are masked during the analysis. Identification of SNPs under neutral selection, and masking recombination events, is a requirement for phylogenetic analysis used for public health surveillance, and for the detection of point source outbreaks. However, assaying the accessory genome by combining the use of short and long read technologies for public health surveillance may provide insight into how recombination events impact on the evolutionary course of STEC O157:H7.

## Introduction

Shiga toxin-producing *Escherichia coli* (STEC) serotype O157:H7 is a zoonotic, foodborne pathogen that can cause severe gastrointestinal disease. Symptoms range from mild self-limiting diarrhea to bloody diarrhea, abdominal pain, nausea, and/or vomiting (Byrne et al., 2015). A subset of patients infected with STEC O157:H7, mainly children and the elderly, are at risk of developing hemolytic-uremic syndrome, a systemic condition associated with renal, cardiac, and neurological complications that can be fatal (Launders et al., 2016). There are approximately 700 case reports of STEC O157:H7 in the United Kingdom each year.^1^ Although case numbers are low compared to *Campylobacter* and *Salmonella*, STEC O157:H7 is regarded as a priority public health pathogen due to the potential for poor clinical outcomes. To mitigate the risks, Public Health England (PHE) operates an enhanced microbiological and epidemiological surveillance program for STEC O157:H7 (Byrne et al., 2015). All fecal specimens from hospitalized patients and from community cases reporting to primary healthcare with symptoms of gastrointestinal disease are tested for STEC O157:H7.^2^

All STEC O157:H7 isolated at hospital laboratories are submitted to the Gastrointestinal Bacteria Reference Unit (GBRU) at PHE, where they are sequenced to derive serotype, Shiga toxin gene (*stx*) profile, and single nucleotide polymorphism (SNP) type (Dallman et al., 2015b; Chattaway et al., 2017). Sequence similarity of pathogen genomes can be used to infer the relatedness between isolates as the fewer SNPs identified between pairs of isolates, the less time since divergence from a common ancestor (Dallman et al., 2018). SNP typing, based on hierarchical single linkage clustering of pairwise SNP distances, is used to detect outbreaks of STEC O157:H7 transmitted *via* the same vehicle and/or from the same source population (Jenkins et al., 2019).

To limit person-to-person transmission, after their symptoms have resolved, children aged five and under, food handlers and healthcare workers infected with STEC O157:H7 are required to submit fecal specimens for further testing to ensure they are no longer shedding the pathogen before returning to nursery school or work.^3^ Microbiological clearance testing for STEC O157:H7 is performed by hospital laboratories, and the submission of serial isolates from the same patient to GBRU is not required. However, occasionally multiple isolates from the same case are submitted to GBRU, where they are sequenced.

In the study, sequencing data from patients for whom more than one isolate of STEC O157:H7 was submitted to GBRU, were reviewed to determine the SNP difference between each isolate pair. One isolate pair from the same patient had a higher than expected SNP difference. The aim of this study was to perform long read sequencing on the two isolates from this isolate pair in order to determine the cause of this discrepancy.

## Materials and Methods

### Short-Read Sequencing (Illumina HiSeq 2500) and Data Processing

Genomic DNA was extracted from cultures of STEC O157:H7 using the QIAsymphony system (Qiagen). The sequencing library was prepared using the Nextera XP kit (Illumina) for sequencing on the HiSeq 2500 instrument (Illumina), run with the fast protocol. FASTQ reads were processed using Trimmomatic v0.27 (Bolger et al., 2014) to remove bases with a PHRED score of <30 from the leading and trailing ends, with reads <50 bp after quality trimming discarded.

### Long-Read Sequencing (Nanopore) and Data Processing

Genomic DNA was extracted and purified using the Qiagen genomic tip, midi 100/G, with minor alterations including no vigorous mixing steps (mixing performed by inversion instead) and elution into 100 μl double processed nuclease-free water (Sigma-Aldrich). Genomic DNA for each extract was quantified using a qubit and the high sensitivity (HS) dsDNA assay kit (Thermo Fisher Scientific), following the manufacturer’s instructions.

Library preparation was performed using the Rapid barcoding kit SQK-RBK004 (Oxford Nanopore Technologies). The prepared libraries were loaded onto a FLO-MIN106 R9.4.1 flow cell (Oxford Nanopore Technologies) and sequenced using the MinION (Oxford Nanopore Technologies) for 24 h.

Data produced in a raw FAST5 format was basecalled and de-multiplexed using Guppy v3.2.6 using the FAST protocol (Oxford Nanopore Technologies) into FASTQ format and grouped in each samples’ respective barcode. The FASTQ files were then de-multiplexed again using Deepbinner v0.2.0 (Wick et al., 2018).

Run metrics were generated using Nanoplot v1.8.1 (De Coster et al., 2018). The barcode and y-adapter from each sample’s reads were trimmed, and chimeric reads split using Porechop v0.2.4 (Wick, 2017a) Finally, the trimmed reads were filtered using Filtlong v0.1.1 (Wick, 2017b) with the following parameters, min length = 1,000 bp, length_weight = 10, keep percent = 90, and target bases = 250 Mbp, to generate approximately 50x coverage of the STEC genome with the longest reads.

### *De novo* Assembly, Polishing, Reorientation, and Annotation

Trimmed and filtered nanopore FASTQ files were assembled Flye v2.4.2 (Kolmogorov et al., 2019), using default parameters. Polishing of the assemblies was performed in a three-step process. Firstly, polishing was initiated using Nanopolish v0.11.1 (Loman et al., 2015) using both the trimmed nanopore FASTQs and FAST5s, for each respective sample accounting for methylation using the --methylation-aware = dam,dcm and --min-candidate-frequency = 0.1. The alignment was generated using Minimap2 v2.17 (Li, 2018) and Samtools v1.7 (Li et al., 2009). Secondly, the polishing was continued with Pilon v1.22 (Walker et al., 2014) using Illumina FASTQ reads as the query dataset with the use of BWA MEM v0.7.17 (Li and Durbin, 2010) and Samtools v1.7 (Li et al., 2009). Finally, Racon v1.2.1 (Vaser et al., 2017) also using BWA MEM v0.7.17 (Li and Durbin, 2010) and Samtools v1.7 (Li et al., 2009) was used with the Illumina reads to produce a final assembly for each sample.

As the chromosome from each assembly was circularized and closed, they were re-orientated to start at the *dnaA* gene (GenBank accession no. NC_000913) from *E. coli* K12, using the --fixstart parameter in Circlator v1.5.5 (Hunt et al., 2015). Prokka v1.13 (Seemann, 2014) with the use of a personalized database (an amino acid FASTA that included all genes annotated in the publicly available samples used in this study) was used to annotate the final assemblies.

### Prophage Detection and Processing

Prophages across both samples were detected and extracted using the updated Phage Search Tool (PHASTER; Arndt et al., 2016). Prophage extraction from the genome occurred regardless of prophage size or quality and any detected prophages separated by less than 4 kbp were conjoined into a single phage using Propi v0.9.0 as described by Shaaban et al. (2016). From here the prophages were manually trimmed to remove any non-prophage genes and were again annotated using Prokka v 1.13 (Seemann, 2014) with the use of a personalized database. The output GenBank (gbk) files were modified to color genes by function.

### Mash and *stx*-Encoding Prophage Phylogeny

Mash v2.2 (Ondov et al., 2016) was used to sketch (sketch length 1,000, kmer length, 21) all extracted *stx*-encoding prophages in samples 818062 and 824422 and all *stx*-encoding prophages found in the publicly available STEC genomes as described by Yara et al. (2020). The pairwise Jaccard distance between the prophages was calculated and a neighbor joining tree computed.

### Variant Calling

Illumina FASTQ reads were mapped to the Sakai STEC O157 reference genome (NC_002695.1) using BWA MEM v0.7.13 (Li and Durbin, 2010) and Samtools v1.1 (Li et al., 2009). Variant positions were identified by GATK v2.6.5 UnifiedGenotyper (McKenna et al., 2010) that passed the following parameters: >90% consensus, minimum read depth of 10, Mapping Quality (MQ) ≥ 30, and imported into SnapperDB v0.2.5 (Dallman et al., 2018). Nanopore FASTQ reads were mapped to the Sakai STEC O157 reference genome (NC 002695.1) using Minimap2 v2.17 (Li, 2018) and Samtools v1.7 (Li et al., 2009). Methylated (5-methyl-cytosine) bases/positions relative to the reference genome were calculated using Nanopolish v0.11.1 (Loman et al., 2015) and masked in the alignment for each sample as described by Greig et al. (2019). The alignment for each sample was used to interrogate discrepant positions identified by SnapperDB previously.

### Selection of Illumina Reads From Variant Positions and Alignment to Nanopore Assembly

Illumina reads covering the list of discrepant SNPs (Table 1) between each of the samples relative to the reference genome were identified using Samtools view v1.7 (Li et al., 2009) and read IDs using Bedtools v2.29.2 (Quinlan and Hall, 2010). These reads were deduplicated and aligned to each respective nanopore assembly and using Bedtools v2.29.2 (Quinlan and Hall, 2010) to identify where each individual read aligned to (Supplementary Material).

**Table 1:** Table showing the variant positions between the two query samples (818062 and 824422) for both sequencing technologies against the reference genome (Sakai).

Pos	REF	VAR	818062 Illumina	824422 Illumina	818062 ONT	824422 ONT	CDS	Locus tag	Product
2196142	G	C	G	C	G	-	L233V	ECs2204	hypothetical protein
2202081	A	G	A	G	A	G	Non coding	-	-
2202082	A	C	A	C	A	C	Non coding	-	-
2202319	C	T	C	T	C	T	Synonymous	ECs2216	putative exonuclease
2202328	C	G	C	G	C	G	Synonymous	ECs2216	putative exonuclease
2202329	C	A	C	A	C	A	H65N	ECs2216	putative exonuclease
2202334	A	G	A	G	A	G	Synonymous	ECs2216	putative exonuclease
2202338	G	A	G	A	G	A	V68I	ECs2216	putative exonuclease
2202343	G	T	G	T	G	T	Synonymous	ECs2216	putative exonuclease
2210582	A	G	A	G	A	-	Non coding	-	-
2210583	T	C	T	C	T	-	Non coding	-	-
2210594	A	G	A	G	A	-	Non coding	-	-
2237846	A	G	A	G	A	G/T	Synonymous	ECs2262	hypothetical protein

POS = position in the reference genome; REF = base in the reference genome at that position; VAR = base in the alignment at that position; A “–” refers to no reads aligned at that position (i.e., 0 depth).

### Data Visualization Tools

All gene diagrams were constructed using Easyfig v2.2.3 (Sullivan et al., 2011). Parsimony trees were visualized and annotated using FigTree v1.4.4 (Rambaut and Drummond, 2018). Dot plots were generated and visualized using Gepard v1.4 (Krumsiek et al., 2007).

### Data Deposition

Illumina FASTQ files are available from National Centre for Biotechnology Information (NCBI) BioProject PRJNA315192 under the following SRA (sequence read archive) accession numbers: 818062; SRR10247133 and 824422; SRR10313636.

Nanopore FASTQ files are available from BioProject PRJNA315192 under the following SRA accession numbers: 818062; SRR12012233 and 824422; SRR12012232.

Assemblies/draft-genomes can be found under BioProject PRJNA315192 under the following accession numbers: 818062; CP058233 (Chromosome), CP058234 (pO157) and 824422; CP058231 (Chromosome), CP058232 (pO157).

## Results

### Analysis of Short-Read Sequencing Data From Isolates From the Same Case

**Figure 1:**
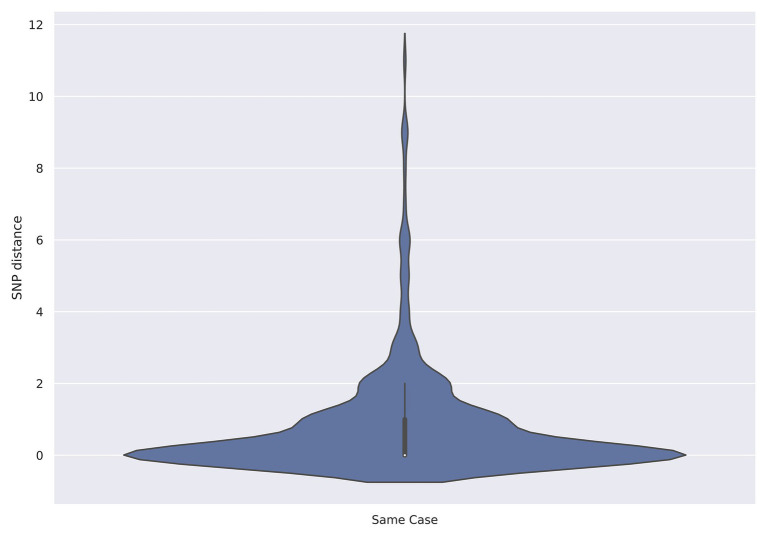
Violin plot showing the distribution of single nucleotide polymorphism (SNP) distances from isolates recovered from the same patient (*N* = 261).

Between July 2015 and December 2019, there were 261 cases where multiple isolates were sequenced from the same person comprising a total of 589 isolates (Supplementary Table). The majority of cases were associated with two isolations (215/261, 82.4%), there were 37/261 (14.2%) cases with three isolates and nine (3.4%) that were linked to between four and nine isolates. The median time between receipts of serial isolates was 6 days, with a minimum of 0 days and a maximum of 133 days. Serial isolates from the same patient fell within five SNPs of each other for 260/261 (99.6%) of the cases (Table 2). The median SNP distance between isolates from the same case was zero SNPs with a maximum of 11 SNPs (Figure 1).

**Table 2:** Table showing total number of Shiga toxin-producing *Escherichia coli* (STEC) cases from 2015 to 2019.

	2015–16	2017	2018	2019	Total
Total number of cases	715^*^	563	607	514	2,399
No. of cases with serial isolation	80	60	54	67	261
No. of isolates from the same case	173	131	130	155	589
Cases with two isolates	71	51	42	51	215
Cases with three isolates	7	8	9	13	37
Cases with four isolates	1	0	1	1	3
Cases with five isolates	0	1	0	2	3
Cases with six isolates	1	0	1	0	2
Cases with nine isolates	0	0	1	0	1

* Numbers for 2016 only.

One case had an isolate pair, which did not cluster into a five SNP single linkage cluster, as identified by comparing the hierarchical SNP profiles (Table 3; Dallman et al., 2018). The case was an 18-month-old female with persistent diarrhea, who had symptoms for more than 10 days prior to presenting to primary healthcare. The first isolate, designated 818062, was from a fecal specimen dated 23rd September 2019 and the second isolate, designated 824422, was from a fecal specimen taken 10 days later on 3rd October 2019. The source of her infection was unknown. Aligned to the reference genome for SNP typing, 818062 and 824422 had 44.66x and 72.37x coverage, respectively. Further analysis of the short-read sequencing data revealed that the two isolates were 13 SNPs different, and that 11 SNPs were located in the same prophage region of the genome (Table 1) occurring in isolate 824422 with respect to the reference genome.

**Table 3:** Table showing *stx* subtype, phage type, receipt date, and SNP address of samples 818062 and 824422.

Molis ID	DOB	Receipt date	Phage type	STX subtype	SNP address
818062	27/02/2018	30/09/2019	PT 34	stx2a stx2c	5.772.1448.3105.3866.5310.6343
824422	27/02/2018	14/10/2019	PT 34	stx2a stx2c	5.772.1448.3105.4913.5281.6387

### Analysis of the Long-Read Sequencing Data From Isolates 818062 and 834422

**Figure 2:**
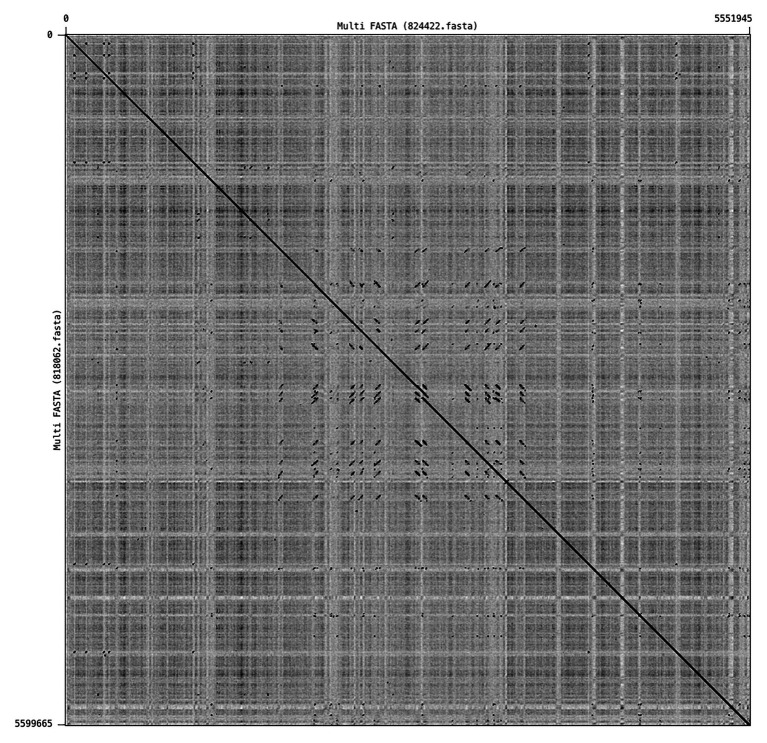
Dot plot showing a whole genome alignment between 818062 (Y-axis) and 824422 (X-axis).

**Figure 3:**
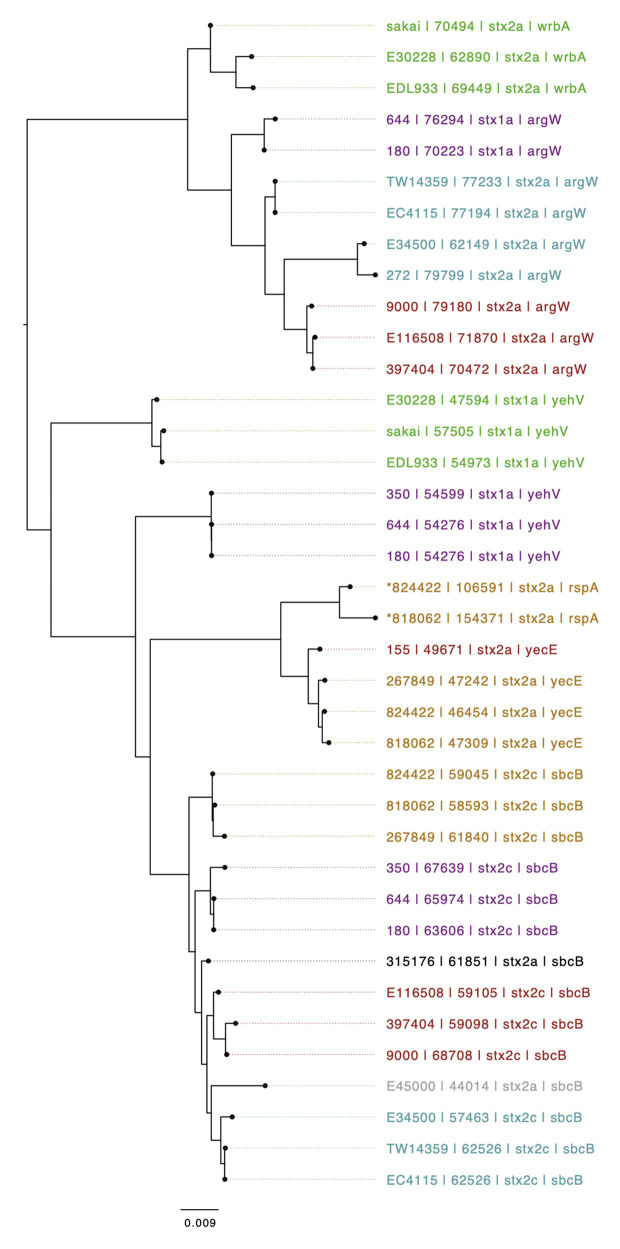
Mid-rooted neighbor joining tree of Shiga toxin-encoding prophages based on Jaccard distance produced from Mash. Strains are annotated as Strain ID, length, *Stx* profile, and Shiga toxin-encoding bacteriophage insertion (SBI). Strains are colored by lineage – Green: Ia, Red: Ic, Blue: I/IIa, Gray: I/IIb, Orange: IIa, Black: IIb, and Purple, IIc. An ^*^indicates if a several prophages are compounded into one i.e., no chromosomal sequence separating them.

The genomic context of the 13 SNP differences identified in the short reads between 818062 and 824422, when compared to the Sakai reference genome, was investigated. Long read sequencing data from isolates 818062 and 824422 was assembled into two contigs for each sample. Each was identified as a single chromosome and the pO157 plasmid. The chromosome size for 818062 was 5,505,066 bp and for 824422 it was 5,457,341 bp, approximately 50 kbp different (Figure 2). The genomes of both isolates 818062 and 824422 comprised 16 prophages each, approximately 13.1 and 12.4% of each chromosome, respectively. In each isolate, three of the 16 prophages encoded *stx*; two had *stx2a* and one had *stx2c* (Figure 3). For the *stx2c*-encoding prophage the Shiga toxin-encoding bacteriophage insertion (SBI) site was *sbcB*. The SBI site of one of the *stx2a*-encoding prophages was *yecE* and the other was *rspA*.

**Figure 4:**
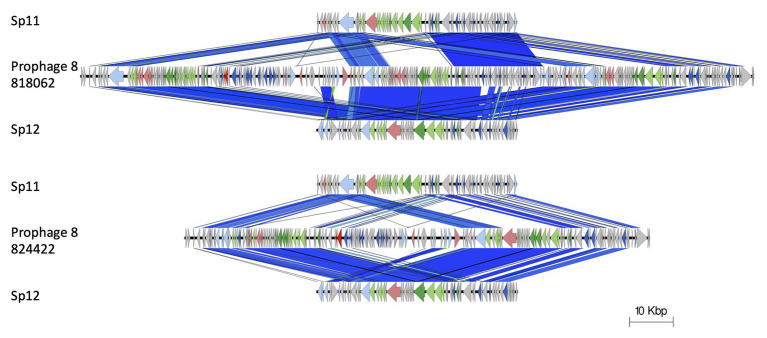
Prophage 8 in 81862 and 824422 has homology to SP11 and SP12 in the Sakai reference genome, with an additional *stx2a*-encoding phage inserted into SP11 component of the compound phage. Arrow indicates gene direction. Recombination/replication genes shown in light blue, *Stx* genes shown in red, regulation associated genes in dark blue. Structure and lysis associated genes shown in light and dark green, respectively, finally gray are hypothetical genes.

**Figure 5:**
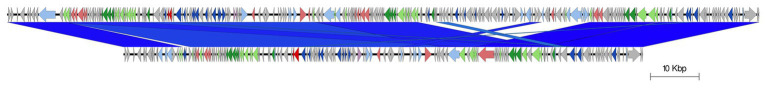
Easyfig alignment of prophage 8 (*stx2a*) for both query samples (818062; top and 824422; below). Arrow indicates gene direction. Recombination/replication genes shown in light blue, *Stx* genes shown in red, regulation associated genes in dark blue. Structure and lysis associated genes shown in light and dark green, respectively, finally gray are hypothetical genes.

The *stx2a*-encoding prophage inserted at *rspA* was a compound prophage, designated prophage 8. In the Sakai reference genome, Sakai prophages (SP) 11 and 12 are co-located in tandem. Prophage 8 in 81862 and 824422 has homology to SP11 and SP12 in the Sakai reference genome, with an additional *stx2a*-encoding phage inserted into SP11 component of the compound phage (Figure 4). Prophage 8 differed in size between isolates 818062 (154,371 bp; position 2,830,861–2,985,232) and 824422 (106,591 bp; position 2,830,714–2,937,305; Figure 5). The size difference was due to homologous recombination resulting in an insertion/deletion event involving a 50 kbp fragment present in prophage 8 in isolate 818062, but absent in isolate 824422.

### Investigation of the Insertion/Deletion Event as the Cause of the Discrepant SNP Difference Between Isolates 818062 and 834422

To determine whether the recombination event in prophage 8 led to the SNP difference between isolates 818062 and 834422, short reads that aligned to the variant positions identified in 824422 relative to the Sakai reference genome for each sequence (shown in Table 1), were identified and mapped back each of their respective long read assemblies. There were 180 non-duplicated reads covering the variant positions relative to the Sakai reference genome for isolate 818062, and 43 non-duplicated reads for isolate 824422.

When mapped back to the long-read sequence assembly of isolate 818062, 167/180 (92.7%) of the short reads from the Illumina data were located within the 50 kbp region of the genome (2,898,039–2,950,379 in 818062) that was not present in isolate 824422 (Supplementary Material). Of the remaining 13/180 (7.2%) reads, seven reads mapped within prophage 8 but outside the region where the recombination event appears to have taken place. The remaining five reads mapped to homologous regions within prophage 1 (*n* = 3 reads) and prophage 11 (*n* = 2 reads).

When aligned to the long read sequence assembly of 824422, where the 50 kbp was absent on prophage 8, 22/43 (51.1%) reads mapped back to homologous regions within prophage 1 (*n* = 18 reads), prophage 3 (*n* = 1 read), prophage 4 (*n* = 1 read), prophage 11 (*n* = 1 read), and prophage 13 (*n* = 1 read). The remaining 21/43 reads (48.9%) mapped back to prophage 8 (Supplementary Material).

The analysis revealed that the 50 kbp fragment present in isolate 818062, but absent in isolate 824422, also had a homologous sequence present in the Sakai reference genome (SP 11 and 12). Therefore, the short reads from the 50 kbp region on isolate 818062 mapped to the corresponding prophage region in the Sakai reference genome with fewer SNP differences than the short reads from paralogous prophage regions in isolate 824422. These short reads from homologous prophage regions in isolate 824422 had less similarity to the homologous region in the Sakai reference genome and therefore a greater number of false positive SNP differences were detected.

To confirm that the absence of the 50 kbp prophage region in isolate 824422 was the reason for the original discrepancy, this prophage region in the Sakai reference genome was masked within the alignment, resulting in zero SNPs difference between isolates 818062 and 824422.

## Discussion

The relatedness between two isolate genomes can be quantified by calculating the number of SNP differences. In general, for clonal bacteria such as STEC O157:H7, the fewer polymorphisms identified between pairs of strains, the less time since divergence from a common ancestor and therefore the increased likelihood that they are from the same source population (Dallman et al., 2018; Jenkins et al., 2019). In this study, we compared SNP profiles from multiple isolations of STEC O157:H7 from the same patient, collected in response to public health guidance that requires patients in risk groups to be excluded from work or nursery school until microbiological clear. All but one isolate pair clustered within a five SNP single linkage cluster indicating that there is little evidence for within host variation of STEC O157:H7 within the time frame required to achieve clearance, as quantified based on the SNP typing method used at PHE (Dallman et al., 2015a).

The investigation into the 13 SNP discrepancies between one of the isolate pairs revealed the cause to be a recombination event within a *stx2a*-encoding prophage resulting in the insertion/deletion of a 50 kbp fragment of the genome. This 50 kbp prophage fragment had a homologous sequence present in the Sakai reference genome (SP11 and SP12), and the short reads from the isolate with the 50 kbp fragment mapped unambiguously to this region in the reference genome. The variants in the isolate without the 50 kbp fragment were attributed to false mapping of the short reads to homologous regions of the reference genome (Greig et al., 2019).

Given the high percentage of prophage in STEC O157:H7 relative to other *E. coli*, and the knowledge that within host variation can occur over a short time frame, we might expect these recombination events to be detected more frequently in isolate pairs from the same patient than the initial analysis in this study suggested (Hayashi et al., 2001; Asadulghani et al., 2009; Eppinger et al., 2011; Shaaban et al., 2016; Greig et al., 2019; Yara et al., 2020). One explanation is that the 18 prophage regions in the Sakai reference genome share similarity, particularly in genes that code for bacteriophage structures (head, tail, and portal genes) and are masked during the analysis using a reference self-mapping strategy (Dallman et al., 2018), as part of the variant calling pipeline reads simulated from the Sakai reference genome are mapped to self. Those regions of the genome where self-mapping was ambiguous, that is, where reads from multiple regions mapped to the same position, or the same reads mapped to multiple positions, are masked from any variant detection (Dallman et al., 2018).

Identification of SNPs under neutral selection, and masking recombination events, is a requirement phylogenetic analysis used for public health surveillance, and for the detection of point source outbreaks of STEC O157:H7 (Dallman et al., 2015b). However, assaying the accessory genome for further genomic characterization offers an additional level of strain of discrimination that may provide insight into the source and/or transmission of an outbreak strain (Cowley et al., 2016). As yet, we have a limited understanding of the rate of change of the recombination taking place in the STEC O157:H7 accessory genome, and the impact this has on the population structure. Combining the use of short and long read technologies for public health surveillance of STEC will improve our understanding of how microevolutionary events and large scale structural variations in the genome contribute to persistence and survival of the pathogen in the environment, colonization and host specificity in the animal reservoir, and the emergence of clinically significant strains (Cowley et al., 2016; Shaaban et al., 2016; Yara et al., 2020).

## Data Availability Statement

Illumina FASTQ files are available from National Centre for Biotechnology Information (NCBI) BioProject PRJNA315192 under the following SRA (sequence read archive) accession numbers: 818062; SRR10247133 and 824422; SRR10313636. Nanopore FASTQ files are available from BioProject PRJNA315192 under the following SRA accession numbers: 818062; SRR12012233 and 824422; SRR12012232. Assemblies/draft-genomes can be found under BioProject PRJNA315192 under the following accession numbers: 818062; CP058233 (Chromosome), CP058234 (pO157) and 824422; CP058231 (Chromosome), CP058232 (pO157).

## Ethics Statement

Ethical review and approval was not required for the study on human participants in accordance with the local legislation and institutional requirements. Written informed consent from the participants’ legal guardian/next of kin was not required to participate in this study in accordance with the national legislation and the institutional requirements.

## Author Contributions

DG performed DNA extraction, library preparation, and Nanopore sequencing. DG performed data processing, genome assembly, correction and annotation. DG created Easyfig diagrams and performed the prophage comparison using Mash with associated scripts designed by TD. DG performed the relatedness analysis including read sub-selection and alignments. TD created violin plot. CJ and DG wrote the original manuscript. CJ, DG, and TD reviewed the manuscript. CJ and TD supervised DG. All authors contributed to the article and approved the submitted version.

### Disclaimer

The views expressed are those of the authors and not necessarily those of the National Health Service, the NIHR, the Department of Health nor PHE.

### Conflict of Interest

The authors declare that the research was conducted in the absence of any commercial or financial relationships that could be construed as a potential conflict of interest.

## Footnotes

^1^https://www.gov.uk/government/publications/escherichia-coli-e-coli-o157-annual-totals

^2^https://www.gov.uk/government/publications/smi-b-30-investigation-of-faecal-specimens-for-enteric-pathogens

^3^https://www.gov.uk/government/publications/shiga-toxin-producing-escherichia-coli-public-health-management
